# Use of aspirin for primary and secondary prevention of cardiovascular disease in diabetic patients in an ambulatory care setting in Spain

**DOI:** 10.1186/1471-2296-8-60

**Published:** 2007-10-17

**Authors:** Antoni Sicras-Mainar, Ruth Navarro-Artieda, Javier Rejas-Gutiérrez, Jaime Fernández-de-Bobadilla, Xavier Frías-Garrido, Rafael Ruiz-Riera

**Affiliations:** 1 Badalona Serveis Assistencials SA, Badalona, Barcelona, Spain; 2 Investigación de Resultados en Salud, Unidad Médica Pfizer, Madrid, Spain

## Abstract

**Background:**

This study was conducted in order to determine the use of aspirin and to assess the achievement of therapeutic targets in diabetic patients according to primary (PP) or secondary prevention (SP).

**Methods:**

This is a retrospective, observational study including patients ≥18 years with diabetes mellitus followed in four primary care centers. Measurements included demographics, use of aspirin and/or anticoagulant drugs, co-morbidities, clinical parameters and proportion of patient at therapeutic target (TT). Descriptive statistics, chi-square test and logistic regression model were used for significance.

**Results:**

A total of 4,140 patients were analyzed, 79.1% (95% confidence intervals [CI]: 77.7–80.5%) in PP and 20.9% (95% CI: 18.2–23.7%) in SP. Mean age was 64.1 (13.8) years, and 49.3% of patient were men (PP: 46.3, SP: 60.7, p = 0.001). Aspirin was prescribed routinely in 20.8% (95% CI: 19.4–22.2%) in PP and 60.8% (95% CI: 57.6–64.0%) in SP. Proportion of patient at TT was 48.0% for blood pressure and 59.8% for cholesterol. Use of aspirin was associated to increased age [OR = 1.01 (95% CI: 1.00–1.02); p = 0.011], cardiovascular-risk factors [OR = 1.14 (95% CI: 1.03–1.27); p = 0.013], LDL-C [OR = 1.42 (95% CI: 1.06–1.88); p = 0.017] and higher glycated hemoglobin [OR = 1.51 (95% CI: 1.22–1.89); p = 0.000] were covariates associated to the use of aspirin in PP.

**Conclusion:**

Treatment with aspirin is underused for PP in patients with diabetes mellitus in Primary Care. Achievement of TT should be improved.

## Background

Arterial hypertension, hypercholesterolemia, smoking, obesity, lack of physical activity and diabetes mellitus represent the main modifiable risk factors for the occurrence of cardiovascular diseases in developed countries [1]. Patients with diabetes mellitus have a cardiovascular risk two to four times higher than the general population. Complications attributable to atherosclerosis are responsible for 70–80% of all deaths in diabetic patients and account for more than 75% of all hospital admissions, causing high rates of disability and health resource utilization [2]. There is currently a clear trend to an increased prevalence, due both to the progressive aging of the population and to the increased frequency of sedentary habits and obesity.

This situation makes institution of drug measures for primary and secondary prevention in patients with cardiovascular disease (CVD) a primary objective. The American Diabetes Association [3] (ADA) recommends use of antiaggregants (low-dose acetyl salicylic acid [ASA], 75–325 mg/day) in all diabetic patients with established CVD (secondary prevention) and in those with no CVD who have a high risk of cardiovascular events (primary prevention) or are over 40 years of age, provided there is no documented contraindication, such as allergy to salicylates, bleeding risk, anticoagulant therapy, recent gastrointestinal bleeding, active liver disease, or the age (to prevent Reye syndrome). These recommendations are based on three studies (ETDRS [4], HOT [5], and the Physicians' Health Study [6] showing that this treatment decreases the incidence of myocardial infarction in people with diabetes. Two of these studies are included in the meta-analysis of the US Preventive Services Task Force [7], which confirmed the decrease in the incidence of myocardial infarction, but found no significant differences in mortality reduction. In this regard, the American Heart Association [8] and the results reported by several meta-analyses [9,10] recommend preventive treatment with ASA in people with a 10-year risk higher than 10%. Despite the fact that various Spanish scientific societies (such as the Catalan Societies of Diabetes, Neurology, Cardiology, Family and Community Medicine, etc.) also support the start of ASA therapy as one of the main cost-effective measures, the scant evidence reported in our setting [11-13] suggest that it is underused. Additional studies conducted in other countries further support the consistency of these results [14-18].

The rationale of the study was to review to what extent these recommendations/guidelines, which are evidence base medicine, are implemented in real world standard condition of care in a primary care setting in Spain. Then, the purpose of this study was to determine the use of ASA for cardiovascular prevention in patients with diabetes mellitus at several health care centers under standard clinical practice conditions, and to ascertain the extent to which some therapeutic control objectives are achieved in primary care setting.

## Methods

### Study design and data extraction

A retrospective, observational study was conducted based on the medical records of patients monitored on an outpatient basis and under standard clinical practice conditions. The study population consisted of people of either sex attending four primary care centers managed by Badalona Serveis Assistencials SA. All patients older than 18 years diagnosed of diabetes mellitus according to the criteria established by the ADA [3] and seen at the center over the past two years (January 2004 and December 2005) were enrolled in the study. Patients with a doubtful diagnosis and patients in whom onset of the disease had occurred less than 3 months before were excluded from the study (n = 186).

Variables recorded included age, sex, social security status (active worker, retired), primary care center identification, time (months) since initial diagnosis of the disease, number of annual visits attended according to the protocol or clinical practice guideline for cardiovascular risk of the centers, number of cardiovascular risk factors, treatment prescribed, and regular use of ASA or other oral antiaggregant and/or anticoagulant drugs (including triflusal, clopidogrel, ticlopidine, warfarin and acenocoumarol). Aspirin regular use by a patient was established when a physician prescribed aspirin for more than 8 months per year. Clinical diagnoses or comorbidities associated to diabetes mellitus were obtained from the International Classification of Primary Care (ICPC-2) [19]. Non-repeated events seen in the population considered included arterial hypertension (K86, K87), hypercholesterolemia (T93: partial), active smoker (P17), obesity (T82); presence of CVD: ischemic heart disease (K74: cardiac ischemia with angina, K75: acute myocardial infarction, K76: coronary ischemia), cerebrovascular accident (K90, K91: transient cerebral ischemia), and peripheral artery disease (K92: intermittent claudicating, Raynaud syndrome, arterial stenosis or embolism); and other co-morbidities: congestive heart failure (K77), renal failure (U99), liver failure (D97), chronic obstructive pulmonary disease (R95: chronic airflow obstruction), depressive syndrome (P76), and benign prostate hypertrophy (Y87). Information was obtained from the computerized clinical records. Legal regulations on data confidentiality were complied with at all times.

### Measurements

The clinical parameters measured included the cardiovascular risk index (Framingham calculation adapted for primary care), body mass index (BMI < 29, kg/m2), systolic (SBP, mmHg) and diastolic blood pressure (DBP, mmHg), total cholesterol (mg/dL), low density lipoprotein cholesterol fraction (LDL-C, obtained by the Friedewald formula [20]), high density lipoprotein cholesterol fraction (HDL-C), and glycated hemoglobin (HbA1c). Some of the established recommendations [3,21] for blood pressure (SBP/DBP < 130/80 mmHg), and LDL-C (<100 mg/dL), see Table 1. Besides, we followed modified criterion to adapt them to a real clinical practice conditions: glycated hemoglobin (<6.5%), total cholesterol (<200 mg/dL) and body mass index (BMI < 29, kg/m2), they were considered as adequate follow-up or target objectives.

**Table 1:** Therapeutic targets in prevention and treatment of cardiovascular disease in diabetes.

*Metabolic control (normal blood glucose)*
- Acceptable: blood glucose < 140 mg/dl and HbA1c < 7%
- Ideal: basal blood glucose < 110 mg/dl and HbA1c < 6%
*Lipid normalization*
- TC < 170 mg/dl and TG < 150 mg/dl
- LDL-C < 100 mg/dl (or non-HDL cholesterol < 130 mg/dl)
- HDL-C > 40 mg/dl.
*Blood pressure control (BP < 130/80 mmHg)*
*Smoking cessation Weight loss (normal weight)*
- Acceptable: BMI < 27 kg/m^2^
- Ideal: BMI < 25 kg/m^2^
*Frequent aerobic physical exercise*
*Other measures*: antiaggregants are under study to show their value in diabetes (use of low-dose ASA is recommended in some subjects with high cardiovascular risk)

HbA1c: glycated hemoglobin; LDL-C: low density lipoprotein cholesterol; HDL-C: high density lipoprotein cholesterol; BMI: body mass index; ASA: acetyl salicylic acid. Source: "Grupo de Trabajo Diabetes mellitus. Diabetes mellitus y riesgo cardiovascular. Recomendaciones del Grupo de Trabajo Diabetes Mellitus y Enfermedad Cardiovascular de la Sociedad Española de Diabetes. Clin Invest Arterioscl 2004;16:74–8".

The last measurement obtained during 2005 was considered in all cases.

### Statistical methods

Data depuration and database refining from mistake or wrong records were carried out in order to obtain good data to run statistical analyses. Personal data were blinded before analysis in order to guarantee proper anonymity of patient's data in accordance with present local regulations with data management and processing. Descriptive statistics (mean, standard deviation and 95% confidence interval) and testing of Gaussian distribution by means of the Kolmogorov-Smirnov test were performed for descriptive purposes. Bivariate analysis using t-test, Man-Whitney test or Chi^2^, as appropriated, were applied to compare main patients' characteristics between primary and secondary prevention. To test for homogeneity of centers, we used an analysis of variance (ANOVA) or Chi^2 ^tests.

Multiple logistic regression models (enter step procedure) were carried out in the total sample, and in the subgroups according with type of prevention. The use of ASA was the dependent variable, and all significant variables observed in the bivariate analysis were incorporated into the models as independent factors. These included mean age (years), sex (male), active workers, time since disease onset (months), number of visits in the annual cardiovascular risk protocol, presence of a cardiovascular history, hypercholesterolemia and active smoking, and achievement of the therapeutic control objectives (dichotomic variables): blood pressure (<130/80 mmHg), total cholesterol (<200 mg/dL), LDL-C (<100 mg/dL) and HbA1c (<6.5%). SPSS version 14.0 for Windows software was used, and a value of p < 0.05 was established as the significant level.

### Ethical considerations

The World Medical Association has developed the Declaration of Helsinki as a statement of ethical principles to provide guidance to physicians and other participants in medical research involving human subjects. The authors show that in the elaboration of the study the basic principles for all medical research and the confidentiality of the data have been respected marked by the law.

## Results

The number of patients with established diagnosis of diabetes mellitus was 4,140 (crude prevalence: 6.4% of the total population), 79.1% (CI: 77.7–80.5%) in primary prevention and 20.9% (CI: 18.2–23.7%) in secondary prevention. Overall, by December 2004, 14.6% of patients followed diet therapy, while 55.2% were receiving oral antidiabetics, 9.7% oral antidiabetics plus insulin, and 20.5% insulin alone.

Table 2 shows the general characteristics of the studied series, the cardiovascular history, and the therapeutic control objectives in patients with diabetes mellitus by primary care center. No differences were seen between centers in cardiovascular events (range, 20.0–22.2%) or cardiovascular history (except for arterial hypertension; range, 49.4–55.8%; p = 0.002), and the percentages seen for hypercholesterolemia, active smoking, and obesity showed a certain homogeneity. The greatest variability between centers was seen in percentage of use of ASA (ranging from 20.0 to 35.3%; p < 0.001), mean patient age (ranging from 62.2 [14.2] to 65.5 [13.5]; p < 0.001), and particularly achievement of therapeutic target objectives. Thus, target blood pressure (<130/80 mmHg) was achieved in 37.4–57.6% of patients (p = 0.002); target total cholesterol (<200 mg/dL) in 42.1–50.7% of patients (p < 0.001); and target LDL-C (<100 mg/dL) in 13.5% to 21.7% of cases (p = 0.000).

**Table 2:** General distribution of patients with diabetes mellitus by primary care center (PCC).

Variables	PCC-1 n = 978	PCC-2 n = 1,486	PCC-3 n = 902	PCC-4 n = 774	p
CVD (events, %)	22.2	20.7	20.0	21.1	ns
Use of ASA (%)	29.1	35.3	20.0	28.2	0.000
Sex (males, %)	46.9	51.5	46.8	50.9	0.041
Mean age (SD), years	62.2 (14.2)	64.1 (13.5)	65.5 (13.5)	64.9 (13.9)	0.000
					
*CV history*					
Hypertension	49.4	51.2	55.8	50.0	0.002
Hypercholesterolemia	35.8	39.5	37.7	36.2	ns
Active smoking	21.6	17.7	18.3	17.2	ns
Obesity (BMI < 29)	48.5	46.9	46.4	43.9	ns
					
*Therapeutic targets*					
BP (<130/80 mmHg)	49.6	37.4	50.1	57.6	0.002
Total cholesterol (<200 mg/dL)	50.6	50.7	41.1	45.0	0.000
LDL-C (<100 mg/dL)	20.6	21.7	13.5	17.1	0.000
Glycated HbA1c (<6.5%)	33.3	34.4	32.0	33.6	ns

Values are given as percentage or mean (SD: standard deviation); p: statistical significance; CVD: cardiovascular disease; ASA: acetyl salicylic acid; CV: cardiovascular; BMI: body mass index; BP: blood pressure (mmHg); LDL-C: low density lipoprotein cholesterol (mg/dL); Hb: hemoglobin.

**Figure 1:**
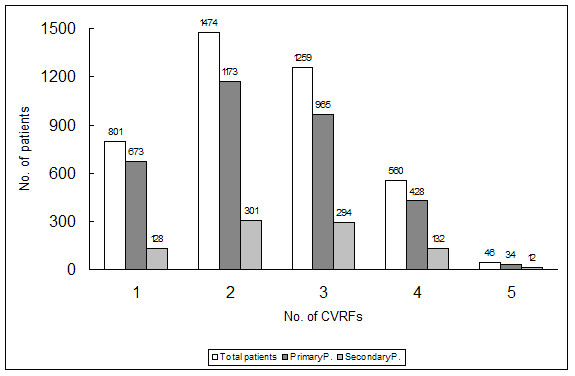
Distribution of the number of cardiovascular risk factors (CVRFs) associated to patients in primary or secondary prevention.

Distribution of the study variables by type of prevention (primary or secondary) is shown in Table 3. Mean patient age was 64.1 (13.8) years, and the values for patients in primary and secondary prevention were 62.3 (14.1) years and 70.9 (10.0) years respectively (p = 0.000). There was a 2.8% primary prevention (PP) patients aged less than 30 years, as compared to none in the secondary prevention (SP) group. Males accounted for 49.3% of the population (PP: 46.3; SP: 60.7; p = 0.000). Of all patients, 29.2% (CI: 27.8%–30.6%) was prescribed in ASA, 20.8% (CI: 19.4%–22.2%) in PP and 60.8% (CI: 57.6%–64.0% in SP; 10.5% of patients were routinely prescribed some other oral antiaggregant or anticoagulant (PP: 5.1%; SP: 30.9%; p = 0.000). In SP, blood pressure (55.9%) and hypercholesterolemia (47.5%) were most prevalent in the history, and the conditions where better therapeutic control objectives were achieved (48.0% and 59.8% respectively; p = 0.000). The distribution of the number of cardiovascular risk factors associated to patients with diabetes mellitus (primary or secondary prevention) is shown in Figure 1. It should be noted that most diabetic patients with no presence of CVD have 2 (35.8%) or 3 (29.5%) cardiovascular risk factors.

**Table 3:** General characteristics of patients with diabetes mellitus by type of cardiovascular prevention (primary, secondary).

Characteristics	Primary n = 3,273	Secondary n = 867	Total n = 4,140	p
Mean age (SD), years	62.3 (14.1)	70.9 (10.0)	64.1 (13.8)	0.000
Sex (males, %)	46.3	60.7	49.3	0.000
SS status (active, %)	37.4	11.2	31.9	0.000
Time since diagnosis (SD), months	43.2 (37.5)	52.6 (49.1)	45.2 (40.4)	0.000
Follow-up per protocol (SD), visits	3.9 (3.2)	4.4 (3.5)	4.0 (3.3)	0.000
				
*CV history*				
Arterial hypertension	49.2	55.9	50.6	0.002
Hypercholesterolemia	34.9	47.5	37.6	0.001
Active smoking	19.3	16.0	18.9	0.026
Obesity (BMI < 29)	47.0	45.2	46.6	ns
				
*Other co-morbidities*				
Heart failure	2.4	8.1	3.6	0.000
Renal failure	1.0	3.2	1.5	0.000
Liver failure	2.8	3.9	3.0	ns
COPD	4.8	12.9	6.5	0.000
Depressive syndrome	14.2	14.6	14.3	ns
Prostatic hypertrophy	4.3	11.1	5.7	0.000
				
*Treatment*				
Acetyl salicylic acid	20.8	60.8	29.2	
Other antiaggregants, OACs	5.1	30.9	10.5	0.000
				
*Clinical parameters*				
Body mass index (SD)	30.3 (5.3)	29.9 (4.9)	30.2 (5.2)	ns
Systolic blood pressure (SD)	135.3 (17.2)	135.4 (17.9)	135.4 (17.4)	ns
Diastolic blood pressure (SD)	78.1 (9.4)	75.7 (9.0)	77.5 (9.3)	0.000
Total cholesterol (SD)	206.9 (41.3)	192.9 (39.9)	203.9 (41.4)	0.000
LDL-C (SD)	133.7 (35.4)	123.7 (35.5)	131.6 (35.6)	0.000
Glycated hemoglobin A1c (SD)	7.2 (1.5)	7.4 (1.4)	7.3 (1.5)	0.014
CVR index (SD)	21.1 (9.1)	25.2 (9.9)	22.1 (9.3)	0.000
				
*Therapeutic targets*				
BP (<130/80 mmHg)	46.1	48.0	46.5	ns
Total cholesterol (<200 mg/dL)	44.1	59.8	47.5	0.000
LDL-C (<100 mg/dL)	16.5	26.9	18.7	0.000
Glycated hemoglobin A1c (<6.5%)	35.2	27.2	33.4	0.001

Values are given as percentage or mean (SD: standard deviation); p: statistical significance; SS: social security; CV: cardiovascular; OACs: oral anticoagulants; CVR: cardiovascular risk; BP: blood pressure (mmHg); LDL-C: low density lipoprotein cholesterol (mg/dL); COPD: chronic obstructive pulmonary disease.

The final regression logistic model (Table 4) showed that patients with controlled-total-cholesterol were significantly associated to the use of ASA in secondary prevention; OR = 1.50; CI: 1.02–2.21; p = 0.039. However, patients on primary prevention, were more likely to use ASA when age increases (OR = 1.01; IC: 1.00–1.02; p = 0.011), show a higher number of cardiovascular risk factors (OR = 1.14; CI: 1.03–1.27; p = 0.013), achieve therapeutic LDL-C targets (OR = 1.42; CI: 1.06–1.88; p = 0.017), and get a poor metabolic control of glycated hemoglobin (OR = 1.51; CI: 1.22–1.89; p = 0.000).

**Table 4:** Correction of general and control variables associated to use of acetyl salicylic acid in primary and secondary prevention.

Correction of logistic model	Primary prevention n = 3,273			Secondary prevention n = 867			Total n = 4,140		
Variables	OR	p	CI	OR	p	CI	OR	p	CI

Age	1.01	0.011	1.00–1.02	0.98	ns	-	1.02	0.000	1.01–1.03
Sex (males)	0.98	ns	-	1.04	ns	-	0.80	0.006	0.68–0.94
Number of CVRFs	1.14	0.013	1.03–1.27	1.03	ns	-	1.16	0.001	1.06–1.26
Total cholesterol	0.89	ns	-	1.50	0.039	1.02–2.21	1.30	0.005	1.08–1.55
LDL-C	1.42	0.017	1.06–1.88	0.83	ns	-	1.29	0.012	1.04–1.62
Glycated HbA1c	1.51	0.000	1.22–1.89	1.07	ns	-	1.47	0.000	1.18–1.79

CVRFs: cardiovascular risk factors; LDL-C: low density lipoprotein cholesterol; OR: odds ratio adjusted by use of acetyl salicylic acid; CI: 95% confidence intervals. Control values incorporated into the final model: total cholesterol (<200 mg/dL), LDL-C (<100 mg/dL), and glycated hemoglobin A1c (<6.5%), p: statistical significance

## Discussion

Studies on the use of acetyl salicylic acid for primary and prevention of cardiovascular disease in adult diabetic patients and their adherence to the recommendations in clinical practice guidelines are heterogeneous in character [11-18], [22-24]. It is important to note that the different methods used for measuring some variables in the reviewed studies make comparisons difficult and requires caution when considering the external validity of results. However, such limitations do not invalidate the current knowledge obtained from such patients, resulting from observations under standard clinical practice conditions in an outpatient setting.

Although explanations may arise when an attempt is made to analyze the general characteristics of patients based on their clinical history (direct correlation between age and arterial hypertension), some degree of uncertainty is generally shown in the care process [25], that may possibly be influenced by the health status of the population and the different existing clinical practice approaches, randomly distributed among the primary care centers reviewed.

In our study, ASA was prescribed by 29.2% of diabetic patients (CI: 27.8–30.6%). However, the proportion increased to 39.4% when use of other antiaggregant or anticoagulant drugs was considered. It should be stressed that in SP the proportion reaches 91.7% of diabetic patients (as compared to 25.9% in PP). Diabetic patients with CVD are mostly older men with a longer time since disease onset and a higher number of associated comorbidities (arterial hypertension and hypercholesterolemia). Moreover, this group has been subject to a more extensive follow-up in the scheduled control visits, and has shown a better achievement of therapeutic goals (except for glycated hemoglobin). It should be noted that a history of CVD clearly suggests to the physician and patient the need for treatment, while the opposite occurs when there is no such history. While, by definition, diabetic patients with no history of cardiovascular disease are considered "primary prevention" patients, the risk for diabetics with no coronary history is in some studies similar to the risk of post-myocardial infarction non-diabetic patients [26]. This fact has caused the control objectives for risk factors in the diabetic population established by scientific associations to be similar to those recommended for secondary prevention patients. These observations demonstrate the potential relationship between monitoring and a better metabolic control [27,28]. An increased awareness and a more decided action by the family physician when faced with a diabetic patient in secondary prevention may also exist [17].

Use of ASA for primary prevention in patients with diabetes mellitus is considered in elderly diabetics with several cardiovascular risk factors, LDL-C therapeutic objectives, and poor metabolic control of glycated hemoglobin. In these patients (with no cardiovascular event), both ASA use and achievement of control objectives can still be improved, particularly taking into account that most primary prevention patients meet criteria for antiaggregant therapy [3,11,22]. Patients and/or healthcare professionals may possibly see regular use of ASA as a non-relevant or second level treatment, as discussed by some authors [16,17] and demonstrated in our study. This suggests that patients with diabetes are not treated as a function of risk, but of the presence of CVD and the degree of metabolic control achieved.

The results obtained, when compared to national studies [11-13], generally show some differences in the characteristics of the diabetic patients studied, possibly attributable to the population profile used, but the rates of ASA underused are similar. Other consulted series show incremental percentages over the years, but lower than desirable values [14-17,24]. Therapeutic control objectives were achieved in similar or slightly higher percentages as compared to other series reviewed [28,29]. It is paradoxical that while various renowned scientific associations [3,8,21] recommend use of ASA or other antiaggregants for primary prevention in diabetic patients older than 40 years, or patients under 21 years of age having some associated risk factor, such advice is not followed in clinical practice. But the most important thing is that a CVD (often angina or infarction) is not prevented [11]. In this regard, the cost of prevention of a cardiovascular event with ASA treatment would be much lower than the costs resulting from hospitalization and sequels, not to speak of the social and personal burden for these patients [2,11].

The efficacy of ASA in clinical trials [4-9,30] contrasts with various pathophysiological studies that would explain why patients with diabetes mellitus may be more resistant to the potential benefits of the drug. Some authors postulate that response to preventive ASA treatment could be different in diabetic patients for several reasons [31]. These would include the fact that diabetes may be a form of resistance to ASA or to low doses of ASA; platelets of these patients could be activated by various mechanisms that would lead to thrombosis, or also because the inflammatory stimuli present in diabetic patients could induce a cyclooxygenase-2 enzyme poorly sensitive to ASA. It has also been postulated that hyperglycemia could generate a significant amount of endoperoxides and thromboxan that would counteract the action of cyclooxygenases [32].

Some limitations of the study require caution in the generalization of the results. Such limitations include the retrospective design of the study, the lack of clinical results in coordination with other care levels (care continuum), and the reasons given for contraindication or intolerance to the drugs, and the lack of measure of ASA with medicinal products excluded from or not financed by the National Health System. The study sample may not be representative of the general primary care population in Spain. However, our results should be interpreted with caution, as further research supporting these results should be conducted in larger patient populations under standard clinical practice conditions. Moreover, the potential specific contraindications for ASA therapy could not be established in our study.

However, the organizational model and clinical action protocols [31] of centers such as the ones participating in the study are very similar, and we agree with several authors [11,12,14] on the need that scientific associations and the healthcare administration design approaches aimed at increasing information and training of healthcare personnel in effective cardiovascular prevention measures, particularly in patients who have not developed any cardiovascular event. The results obtained should be of use when considering, based on the available evidence [3,33], whether treatment should be started with ASA or other antiaggregant, when indicated, as it seems obvious that treatment with low-dose ASA is one of the most favorable cost-effective measures in cardiovascular prevention in diabetic patients [3,22,34-37]. Future research in diabetic patients should explore monitoring of preventive measures, effectiveness of the optimum recommended doses [38], or the specific reasons for poor patient compliance.

## Conclusion

In our study treatment with ASA was underused for primary cardiovascular prevention in patients with diabetes mellitus. Achievement of the established therapeutic objectives should be improved. New representative studies under standard clinical practice conditions would be required.

## Competing interests

The author(s) declare that they have no competing interests.

## Authors' contributions

Each author participated sufficiently in the work to take public responsibility for appropriate portions of the content. All authors read and approved the final manuscript. ASM: conceived of the study, and participated in its design and coordination.

## Pre-publication history

The pre-publication history for this paper can be accessed here:


